# Neurophysiological effective network connectivity supports a threshold-dependent management of dynamic working memory gating

**DOI:** 10.1016/j.isci.2024.109521

**Published:** 2024-03-18

**Authors:** Julia Elmers, Shijing Yu, Nasibeh Talebi, Astrid Prochnow, Christian Beste

**Affiliations:** 1 Cognitive Neurophysiology, Department of Child and Adolescent Psychiatry, Faculty of Medicine, TU Dresden, Dresden, Germany

**Keywords:** Neuroscience, Cognitive neuroscience

## Abstract

To facilitate goal-directed actions, effective management of working memory (WM) is crucial, involving a hypothesized WM "gating mechanism." We investigate the underlying neural basis through behavioral modeling and connectivity assessments between neuroanatomical regions linked to theta, alpha, and beta frequency bands. We found opposing, threshold-dependent mechanisms governing WM gate opening and closing. Directed beta band connectivity in the parieto-frontal and parahippocampal-occipital networks was crucial for threshold-dependent WM gating dynamics. Fronto-parahippocampal connectivity in the theta band was also notable for both gating processes, although weaker than that in the beta band. Distinct roles for theta, beta, and alpha bands emerge in maintaining information in WM and shielding against interference, whereby alpha band activity likely acts as a “gatekeeper” supporting processes reflected by beta and theta band activity. The study shows that the decision criterion for WM gate opening/closing relies on concerted interplay within neuroanatomical networks defined by beta and theta band activities.

## Highlights

• WM gate opening and closing are opposing threshold-dependent mechanisms
• Beta Band network connectivity most important for WM gating dynamics
• Higher beta network connectivity during WM gate closing than gate opening
• Alpha Band network connectivity likely acts as a “gatekeeper” for gate opening

## Graphical abstract

**Figure:**
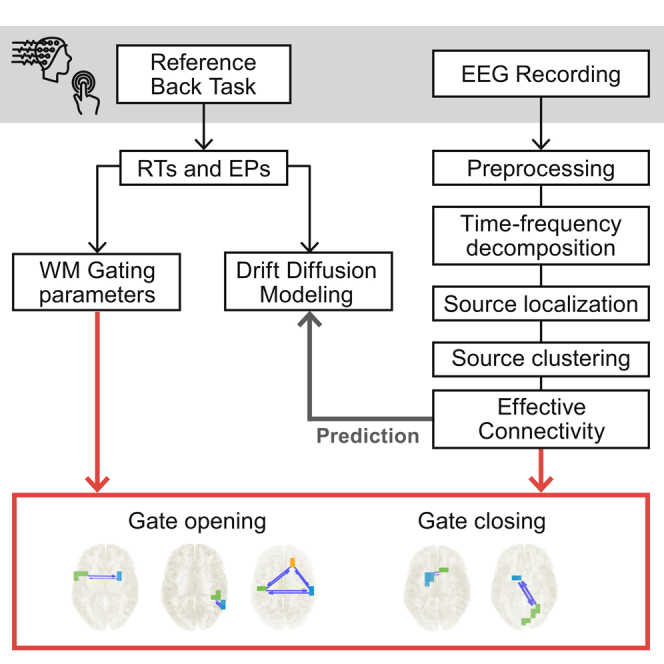

## Introduction

Working memory (WM) processes include the updating of relevant information as well as the shielding of its content from irrelevant input for the successful maintenance of information.^1^ The dynamic management of these opposing processes is crucial for goal-directed acting, but there is an ongoing debate on how the WM manages these processes. A prominent theory involves the idea of a “gating mechanism” to accomplish this. As long as the gate is closed, information within the WM is maintained, whereby an open gate allows the updating of information.^2^^,^^3^^,^^4^ The closed state is assumed to reflect the default WM state, which allows the maintenance of representation through shielding WM from distraction.^5^^,^^6^ So far, several studies have showed WM gate opening and closing effects and delineated some underlying neural principles,^7^^,^^8^^,^^9^^,^^10^^,^^11^^,^^12^ which are triggered by useful or distracting perceptual information, respectively.^13^ However, it is still unclear how the decision to open or close the WM gate is implemented on a computational neural level.

The dynamics of WM gate opening and closing reflect an arbitration between opposing states. Such an arbitration has often been framed as a metacontrol problem, reflecting a dynamic, requirement-sensitive balancing of different subprocesses.^14^^,^^15^^,^^16^ This requirement-sensitive balancing refers to the basis of how the decision is reached when to open or to close the gate. Shifting from one state to another has frequently been conceptualized in “attractor landscapes,”^16^ whereby attractor states can be metaphorically seen as magnets of the system; i.e., they are stable points of the system. Therefore, a certain force (or threshold) needs to be reached to get from one magnet/state (i.e., gate opening) to another magnet/state (i.e., gate closing) of the system. Notably, this “threshold” idea has also been used to frame neurobiological processes within the prefrontal cortex in general and WM in more particular.^17^^,^^18^^,^^19^ From a conceptual perspective, it is thus conceivable that a thresholding mechanism is the central mechanistic element behind the decision when to open or to close the WM gate (i.e., how much evidence needs to be accumulated). Using drift-diffusion model (DDM) it is possible to delineate distinct computational principles underlying decisions that become apparent in overt behavior.^20^^,^^21^ In particular, the so-called “decision threshold/response boundary” parameter (a)^20^ reflects a threshold to which evidence for one decision or another must be collected before a decision is made. If WM gate opening and closing processes reflect threshold-dependent dynamics, especially the DDM parameter (a) should reflect WM gate opening and closing processes. That is, especially the DDM parameter (a), but not or less so the other DDM parameters, should be predictable on the basis of neurophysiological processes associated with WM gate opening and closing.

Evidence suggests that theta, alpha, and beta frequency bands are involved in modulating WM processes.^4^^,^^10^^,^^13^ Theta activity (4–7 Hz) is relevant during the sequential coding of WM items,^22^ and this sequential coding requires the updating of WM content that is likely regulated by an input-gating mechanism.^1^^,^^2^^,^^9^^,^^10^ Consequently, theta activity is linked to and may predict threshold-dependent WM gate opening and closing processes. However, especially when it comes to the maintenance of information in prefrontal cortices (reflecting WM gate closing),^23^ beta activity (13–30 Hz) is also central to consider. Beta activity has been associated with the monitoring of a “status quo.”^24^ which involves the maintenance of information.^25^ More recently, it has been proposed that beta activity reflects changes from active to latent to re-activated states of WM content.^26^ While the functional relevance of beta activity is still debated,^25^^,^^26^ the available evidence nevertheless suggests that beta activity may also be a relevant factor supporting threshold-dependent dynamics of WM gating. Lastly, alpha band activity (8–12 Hz) may also be relevant to consider. This is mainly because alpha activity likely supports mechanisms enabling a selection what information is processed more in depth^27^ and gains access to memory system (including WM). In the current study we examine the relative relevance of each of these frequency bands as neural processes underlying a threshold-dependent dynamics of WM gating.

However, when considering the neurophysiological dynamics, it is relevant to consider that WM processes reflect a prime example of distributed neural processes.^12^^,^^28^ It has been outlined that there is distributed, content-specific WM activity in brain regions such as the lateral prefrontal cortex and the posterior parietal cortex as well as in ventral visual stream regions, because of a division of labor.^28^ Sensory regions likely encode low-level details of WM information, while more abstract information unrelated to a specific modality is processed in parietal and prefrontal regions.^28^ It is this distributed processing of WM information that gives rise to the stability of WM content^28^; that is, every cortical region involved in the processing of information can produce activity needed to maintain information in WM. In particular, it has been shown that the directed communication between functional neuroanatomical structures (i.e., prefrontal to parietal areas) seems to be relevant for the modulation of top-down control in WM processes.^29^^,^^30^^,^^31^ Especially this top-down control is needed for the gate closing mechanism, which enables the shielding of information thus allowing WM maintenance. Therefore, it is likely that fronto-parietal network-like dynamics in aforementioned frequency bands well reflect WM gate opening and closing processes on a computational neural level. To delineate directed communication between cortical structures associated with WM gate opening and closing processes, we use non-linear Causal Relationship Estimation by Artificial Neural Network (*nCREANN*).^32^ In sum, we suggest that neural network dynamics in theta, beta, or alpha band activity modulate WM gating processes. More importantly, we assume that the architecture of directed communication between cortical regions (as examined through nCREANN) might support the idea of a threshold-dependent mechanism for gate opening and closing (i.e., by predicting the thresholding parameter [a] in the DDM).

## Results

We derived three factors from the reference-back task for the subsequent ANOVAs, namely *Switching* (switch vs. nonswitch), *Trial Type* (reference vs. comparison), and *Matching* (match vs. mismatch).

In this paradigm, reaction times (RTs) describe the time between stimulus presentation and reaction (i.e., key press), while error percentages (EPs) refer to response accuracies in the three factors (i.e., *Switching*, *Trial Type*, and *Matching)*. For all analyses, we will refer to switch costs when RTs, EPs, or DDM parameters differ between switch and nonswitch trials (switch-nonswitch). Further, the term “type costs” is used to describe differences between trial types (reference-comparison). Lastly, “match costs” will refer to differences between match and mismatch trials (mismatch-match).

For the calculation of the WM gating parameters, not only the trial type is needed but also the information, if a switch between the two trial types is evident (e.g., from reference to comparison and vice versa) or not (e.g., from comparison to comparison or from reference to reference). The latter is a nonswitch trial. Accordingly, the combination of the factors *Switching* and *Trial Type* results in four different conditions (i.e., switch-reference, nonswitch-reference, switch-comparison, nonswitch-comparison), which are used to calculate gate opening and gate closing (see Equation 1). The factor *Matching* is not considered when calculating the gating parameters:

Gateopening=switch−reference−nonswitch−reference

(Equation 1) Gateclosing=switch−comparison−nonswitch−comparison

Gate opening and closing values can therefore be calculated for all parameters (e.g., RTs, EPs, or DDM parameters).

### Behavioral results

#### RTs

**Figure 1:**
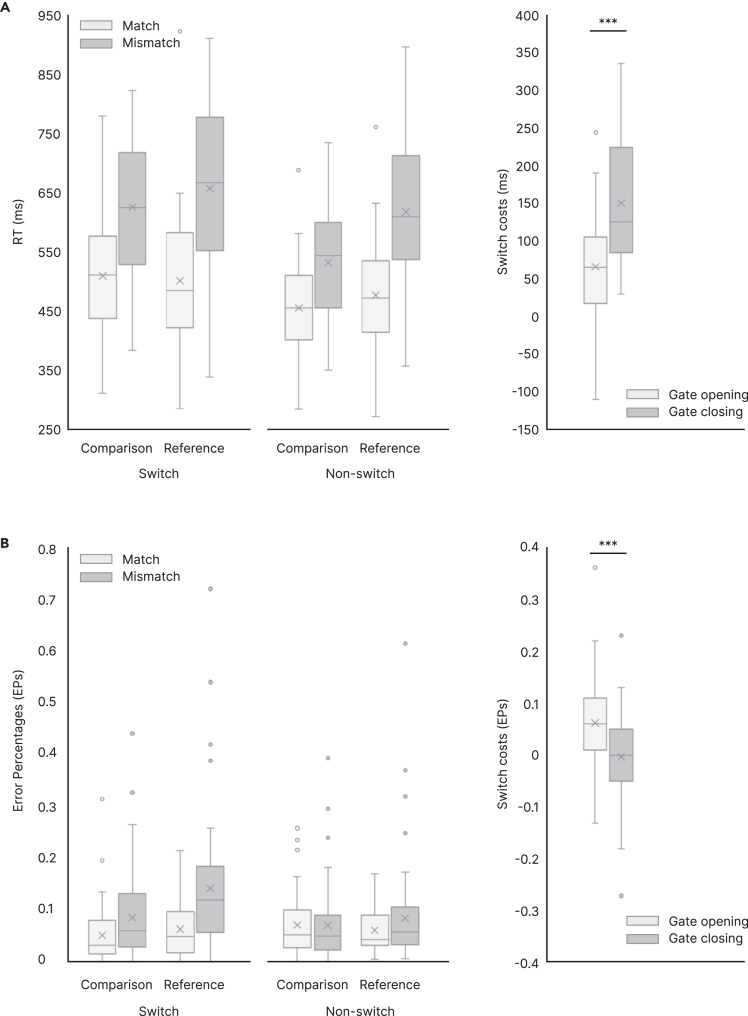
Behavioral results Boxplots of behavioral results of the reference-back task with mean (X) and outliers outside of the 1st and 3rd quartile (left) and gating conditions (right). (A) 3-way ANOVA between Trial type (comparison vs. reference), Switching (switch vs. nonswitch), and Matching (match vs. mismatch) for RTs (left) and RT switching costs in gate opening and closing (right). (B) 3-way ANOVA between Trial type, Switching, and Matching for EPs (left) and EP switching costs in gate opening and closing (right). ∗∗∗p < 0.001. For clarity, indicators of significance were not shown for RTs and EPs (see main text for ANOVA results).

Figure 1A shows the behavioral data for RTs. For RTs, we found all main effects of a repeated-measures ANOVA were significant (*Switching*: F(1,60) = 210.98, p < 0.001, $\eta_{p}^{2}$ = 0.779; *Trial Type*: F(1,60) = 93.42, p < 0.001, $\eta_{p}^{2}$ = 0.609; *Matching*: F(1,60) = 317.29, p < 0.001, $\eta_{p}^{2}$ = 0.841) with slower RTs in switch vs. nonswitch trials, reference vs. comparison trials, and mismatch vs. match trials. Further, the ANOVA revealed significant interactions of *Switching×Trial Type* (F(1,60) = 48.03, p < 0.001, $\eta_{p}^{2}$ = 0.445), *Switching×Matching* (F(1,60) = 36.44, p < 0.001, $\eta_{p}^{2}$ = 0.378), and *Trial Type×Matching* (F(1,60) = 48.65, p < 0.001, $\eta_{p}^{2}$ = 0.448). Importantly, the 3-way-interaction including all factors *Switching×Trial Type×Matching* (F(1,60) = 8.72, p = 0.004, $\eta_{p}^{2}$ = 0.127) was significant. When resolving this interaction by the factor *Switching*, we found a significant interaction between *Trial Type×Matching* in switch trials (F(60,1), p < 0.001, $\eta_{p}^{2}$ = 0.235) and in nonswitch trials (F(60,1), p < 0.001, $\eta_{p}^{2}$ = 0.573). In the nonswitch condition, *post hoc* paired t tests established significantly longer RTs in reference trials than in comparison trials in both the match (t(60) = 4.10, p < 0.001, d = 0.525) and the mismatch conditions (t(60) = 14.70, p < 0.001, d = 1.882). Also in the switch condition, RTs were significantly longer in reference trial than those in comparison trials in the mismatch condition (t(60) = 3.79, p < 0.001, d = 0.485). However, crucially, in match trials, the direction of the RT difference between trial types was opposite, with longer RTs in comparison than in reference trials (t(60) = −2.04, p = 0.023, d = −0.261).

Regarding the gating parameters, which derive from the factors *Switching* and *Trial Type* by subtracting nonswitch-reference from switch-reference trials (i.e., gate opening) and nonswitch-comparison from switch-comparison trials (i.e., gate closing) regardless of the matching condition, we found longer RTs in gate closing compared to gate opening (t(60) = 6.93, p < 0.001, d = 0.887).

#### EPs

Figure 1B shows the behavioral data for EPs. For EPs, main effects of a repeated-measures ANOVA were also significant (*Switching*: F(1,60) = 33.69, p < 0.001, $\eta_{p}^{2}$ = 0.360; *Trial Type*: F(1,60) = 20.87, p < 0.001, $\eta_{p}^{2}$ = 0.258; *Matching*: F(1,60) = 19.59, p < 0.001, $\eta_{p}^{2}$ = 0.246) with higher EPs in switch vs. nonswitch, reference vs. comparison, and mismatch vs. match trials. Again, the significant two-way interactions *Switching×Trial Type* (F(1,60) = 14.86, p < 0.001, $\eta_{p}^{2}$ = 0.199), *Switching×Matching* (F(1,60) = 56.52, p < 0.001, $\eta_{p}^{2}$ = 0.437), and *Trial Type×Matching* (F(1,60) = 5.98, p =0 .017, $\eta_{p}^{2}$ = 0.091) could be established. For the interaction *Switching×Trial Type*, *post hoc* analysis revealed significant switch costs in reference (t(60) = 6.45, p <0 .001, d = 0.826) but not in comparison trials (t(60) = 0.188, p = 0.851), and “type costs” in switch (t(60) = 5.751, p <0 .001, d = 0.736) but not in nonswitch trials (t(60) = 0.174, p = 0.863). Regarding the interaction *Switching×Matching*, significant switch costs were observed in both match (t(60) = −2.257, p = 0.028, d = −0.289) and mismatch (t(60) = 7.493, p <0 .001, d = 0.959) trials, but there were significant “match costs” only in switch (t(60) = 6.042, p <0 .001, d = 0.774) but not in nonswitch (t(60) = 1.114, p = 0.270) trials. For the interaction *Trial Type×Matching*, significant “match costs” were found in reference (t(60) = 4.352, p <0 .001, d = 0.557) but not in comparison (t(60) = 1.850, p = 0.069) trials, and significant “type costs” appeared in mismatch (t(60) = 3.564, p <0 .001, d = 0.456) but not in match (t(60) = 0.068, p = 0.946) trials. The interaction of *Switching×Trial Type×Matching* did not reach significance (F(1,60) = 3.40, p = 0.070).

When looking at the gating processes, there was a significant difference in EPs (t(60) = −3.855, p < 0.001, d = −0.494) between gate closing (0.2 ± 7.4%) and gate opening (6.2 ± 7.5%).

### Drift-diffusion modeling

Based on the trial-by-trial accuracy and RT, a DDM was employed to examine the underlying parameters of the selection process of the response. The thresholds of the DDM were defined as “match” (upper threshold) and “mismatch” (lower threshold), and only correct trials were included in the analysis. The boundary separation (a), drift rate (v), and non-decision time (t0) were estimated as a function of the “switch” (nonswitch vs. switch trials) and “trial type” (comparison vs. reference trials) conditions and will be compared statistically between the conditions in the following.

**Figure 2:**
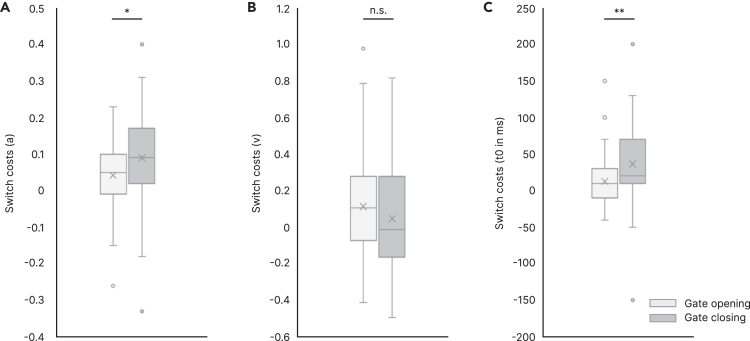
DDM results Boxplots of switch costs in gate opening and closing of DDM parameters, namely (A) boundary separation (a), (B) drift rate (v), and (C) non-decision time (t0). Switch costs were significantly larger for boundary separation (a) and non-decision time (t0) in gate closing compared to opening. ∗p < 0.05, ∗∗p = 0.001, n.s. = non-significant.

Regarding the boundary separation parameter (a), a repeated-measures ANOVA with the factors *Switching* and *Trial Type* revealed significant main effects of both factors (*Switching*: F(1,60) = 49.31, p < 0.001, $\eta_{p}^{2}$ = 0.451; *Trial Type*: F(1,60) = 44.73, p < 0.001, $\eta_{p}^{2}$ = 0.427). The boundary separation was larger in switch (0.93 ± 0.14) than in nonswitch trials (0.86 ± 0.12), and also larger in reference (0.93 ± 0.13) than in comparison trials (0.86 ± 0.12). Importantly, the interaction of *Switching×Trial Type* was significant (F(1,60) = 6.43, p = 0.014, $\eta_{p}^{2}$ = 0.097): boundary separation was significantly larger in gate closing (i.e., the switch costs in comparison trials; 0.09 ± 0.12) than gate opening (i.e., the switch costs in reference trials; 0.04 ± 0.09; t(60) = −2.54, p = 0.014, d = −0.325) (Figure 2A).

Regarding the drift rate (v), a repeated-measures ANOVA established significant main effect of *Switching* (F(1,60) = 9.46, p = 0.003, $\eta_{p}^{2}$ = 0.136), showing a larger drift rate in switch (0.09 ± 0.20) than in nonswitch trials (0.01 ± 0.13). The main effect of *Trial Type* (F(1,60) = 1.52, p = 0.223) and the interaction of *Switching×Trial Type* (F(1,60) = 2.03, p = 0.159) did not reach significance. Therefore, there was no significant difference between gate opening (0.12 ± 0.27) and closing (0.04 ± 0.30) in the (v) parameter (t(60) = 1.425, p = 0.159) (Figure 2B).

With respect to the non-decision time (t0), a repeated-measures ANOVA showed a significant main effect of *Switching* (F(1,60) = 33.96, p < 0.001, $\eta_{p}^{2}$ = 0.361), indicating that the non-decision time was longer in switch (356 ± 57 ms) than in nonswitch trials (332 ± 49 ms). Crucially, the interaction of *Switching×Trial Type* was significant (F(1,60) = 11.15, p = 0.001, $\eta_{p}^{2}$ = 0.157), showing that RT costs were significantly greater in gate closing (35 ± 49 ms) than gate opening (11 ± 32 ms; t(60) = 3.340, p = 0.001, d = 0.428) (Figure 2C). The main effect of *Trial Type* was not significant (F(1,60) = 1.07, p = 0.305).

### Neurophysiological results

#### Oscillatory signatures of WM gating processes

**Figure 3:**
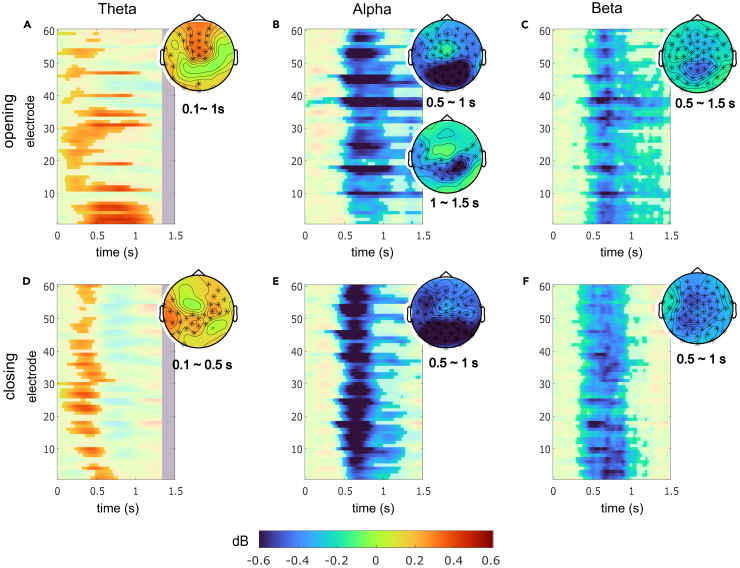
Cluster-based permutation test Results of cluster-based permutation test in gate opening (A‒C) and closing (D‒F) across all frequency bands (theta, alpha, and beta) and electrodes showed significant differences in all gating conditions and frequency bands. Color indicates the decibel value of frequency powers. For time-electrode matrices, time points and electrodes with insignificant gating effect are obscured by transparency. The blank areas in plots A and D represent missing data due to edge effect of time-frequency decomposition. For topographies, electrodes with significant gating effect are marked with ‘∗’.

The cluster-based permutation tests revealed the WM gating effects on all frequency bands of interest (Figure 3). The WM gating effect was calculated as a difference between switch and nonswitch trials (i.e., switch cost). Specifically, the WM gate opening was reflected by theta activity at the early phase (between around 100 and 1,000 ms). That is, theta activity was higher in the switch-reference trials than the nonswitch-reference ones, particularly around the frontocentral electrodes (Figure 3A). The WM opening effect was indicated by alpha activity around 500 to 1,000 ms at almost all electrodes and around 1,000 to 1,500 ms at electrodes of the central site. For both time windows, alpha activity was stronger in the nonswitch-reference trials than the switch-reference trials, suggesting a suppression of alpha activity while opening the gate (Figure 3B). The WM gate opening effect was evident from 500 ms till 1,500 ms at almost all electrodes and represented as a suppression of beta activity (Figure 3C), resembling the alpha activity during WM gate opening. For the WM gate closing process, the significant effect was found to be in a similar pattern to the WM opening but rather shorter timewise. That is, higher theta activity was detected in the switch-comparison trials compared with nonswitch trials from around 100 to 500 ms at electrodes of the left central site and the right frontal site (Figure 3D), while lower alpha and beta activity was observed in the switch-comparison trials than the nonswitch-comparison trials between 500 and 1000 ms at almost all electrodes (Figures 3E and 3F).

After evaluating that the substantial differences at the sensor level, linearly constrained minimum variance (LCMV) beamforming analyses with subsequent voxel clustering using the density-based spatial clustering of applications with noise (DBSCAN) algorithm were carried out for both conditions and all three frequency bands (for detailed description, see STAR Methods section). Table 1 shows the resulting regions with their corresponding Brodmann area.

**Table 1:** Cluster labels and corresponding Brodmann areas for the three frequency bands from the DBSCAN algorithm

Condition	Frequency band	Labels of figures	Neuroanatomical regions
Gate opening	Theta	PHG	right parahippocampal gyrus (BA 27, 28, 36, 37)
		IFG	left inferior frontal gyrus (BA 44)
	Alpha	MTG	right middle temporal gyrus (BA 21)
		IPL	right inferior parietal lobule (BA 40)
	Beta	SMG	right supramarginal gyrus (BA 40)
		PoG	left postcentral gyrus (BA 4)
		PrG	left precentral gyrus (BA 1,2,3)
		SFG	right superior frontal gyrus (BA 8)
Gate closing	Theta	PHG	left parahippocampal gyrus (BA 27, 28, 36, 37)
		SMA	right supplemental motor area (BA 6)
	Alpha	OFC	orbitofrontal cortex (BA 8, 10, 11, 12, 46, 47)
	Beta	PHG	left parahippocampal gyrus (BA 27, 28, 36, 37)

#### Linear and non-linear connectivity

The time courses of the source voxels within each cluster (estimated by DBSCAN) were averaged to obtain one signal for each region representing the activity of that cluster. These average time series were utilized for the subsequent connectivity analysis with the nCREANN method in the theta, alpha, and beta frequency bands. For gate closing, the alpha band was left out of the analysis because there was only one brain region showing activity modulations (see Table 1).

**Figure 4:**
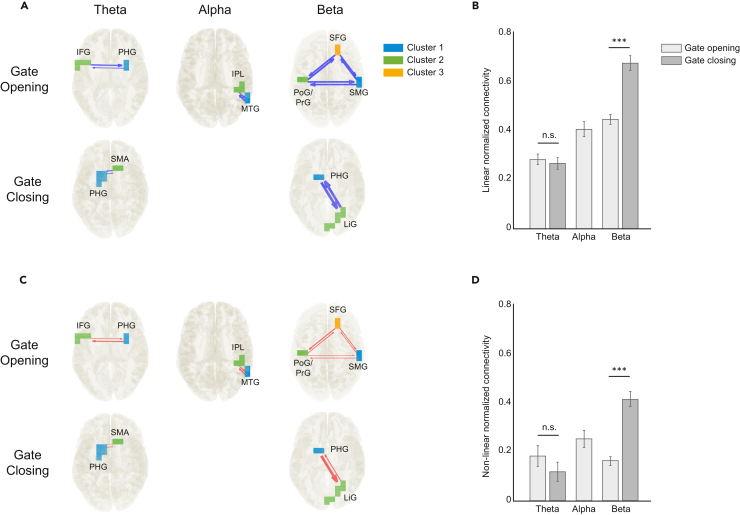
Averaged linear and non-linear connectivity Connectivity patterns between clusters (left) and averaged normalized cluster connectivity strength for all frequency bands (right) are shown for linear (A, B) and non-linear (C, D) effective connectivity, for both gate opening and closing. Thicker arrows indicate stronger effective connectivity from one region to another, while thinner arrows indicate weaker connectivity values. Since the DBSCAN algorithm only showed one cluster during gate closing in alpha band activity, no effective connectivity was calculated. Error bars indicate standard error of the mean (SEM). ∗∗∗p < 0.001, n.s. = non-significant.

In Figure 4, the linear (top-blue) and non-linear (bottom-red) connectivity patterns are displayed for the averaged values across all subjects for gate opening and gate closing. As can be seen, the beta frequency band exhibits very strong linear (Figure 4A) and non-linear (Figure 4C) connections in comparison to theta and alpha frequency bands (i.e., thickness of arrows). Further, Figure 4 illustrates the average strength of connectivity among clusters for each frequency band in each WM gating process. Comparing the WM gate opening and closing processes, it is shown that a significant difference between them was observed only for the beta band activity (Figures 4B and 4D). That is, stronger connectivity was observed in the WM gate closing than gate opening process for both linear (connectivity in WM gate opening: 0.45 ± 0.15; in gate closing: 0.68 ± 0.23; t(62) = 5.79, p ≤ 0.001, d = 0.85) and non-linear connectivity (connectivity in WM gate opening: 0.17 ± 0.13; in gate closing: 0.42 ± 0.24; t(62) = 6.78, p ≤ 0.001, d = 0.85). However, we did not find significant differences between WM gating in the theta band for either linear connectivity (connectivity in WM gate opening: 0.29 ± 0.15; in gate closing: 0.27 ± 0.17; t(62) = 0.67, p = 0.504, d = 0.09) or non-linear connectivity (connectivity in WM gate opening: 0.18 ± 0.33; in gate closing: 0.12 ± 0.30; t(62) = 1.12, p = 0.266, d = 0.14).

**Figure 5:**
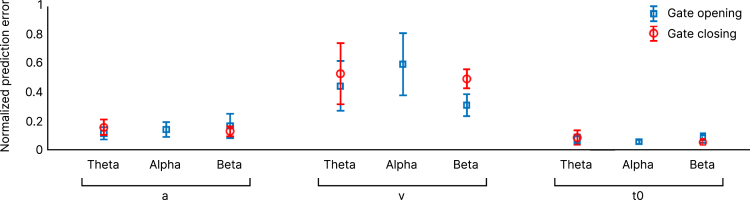
Non-linear regression analysis Results of non-linear regression analysis with normalized prediction errors shows that DDM parameters boundary separation (a) and non-decision time (t0) can be better estimated by effective network connectivity than drift rate (v), with no difference in predictive power between gate opening and closing in (a) and (t0). However, within the beta frequency band, (v) could be significantly better estimated in gate opening compared to gate closing. Error bars indicate a 95% confidence interval (CI) with overlapping intervals showing non-significant differences.

Furthermore, Figure 5 displays the normalized estimation error (scaled to the average absolute value of each parameter) with the 95% confidence bounds shown as the error bars for theta, alpha, and beta frequency bands in gate opening and gate closing. This prediction error is a goodness of fit measure assessing how well the non-linear regression model (a trained network) generalizes to each new, unseen behavioral parameter based on the connectivity values. As seen in Figure 5, in all frequency bands (a) and (t0) could be better predicted (with smaller error values) than (v) by effective network connectivity. However, only for (v) in the beta frequency band, there was a significant difference (confidence interval [CI] > 0.95) between the gating conditions in the prediction error. We found no significant difference in the estimation of the DDM parameters when using the neuronal activity index (NAI) instead of effective network connectivity (see Figure S1). Figure S2 shows the scatterplots of the linear and non-linear connectivity and DDM with the connectivity in the theta, alpha, and beta frequency bands. It is shown that the distribution of the connectivity/DDM parameters is complex and non-linear. Therefore, an artificial neural network approach to perform a non-linear regression between the connectivity information and the DDM parameters in the two gating conditions was used.

## Discussion

The dynamic management of updating and maintaining information in WM is central to adapt behavior during goal-directed actions. Yet, how this dynamic management is implemented on a neural level is still a matter of debate. The current study investigated the underlying mechanism supporting a dynamic management of WM gate opening and closing by means of computational behavioral modeling in combination with analyses of effective connectivities in different frequency bands to capture neural network dynamics.

On a behavioral level, participants committed fewer errors and showed longer RTs in gate closing than opening, replicating previous research.^7^^,^^8^^,^^9^^,^^10^^,^^11^ As mentioned earlier, gate closing and opening represent switch costs from a reference to a comparison trial and vice versa. The DDM of the behavioral data provided further insights into the computational mechanisms. Our data showed no significant differences in the drift rate (*v*) between the gating processes but asymmetric switch costs with a larger boundary separation (*a*) and longer non-decision times (t0) during gate closing compared to opening. Higher switch costs have been repeatedly shown when switching to an easier task.^13^^,^^33^^,^^34^^,^^35^ Possible explanations for this are higher task-priming effects in the difficult trial (i.e., reference)^34^ and a greater need to inhibit the easy task set due to its higher base-level activation leading to increased switch costs due to inhibition carryover.^35^ Moreover, higher switch costs in gate closing might arise from an updating dilemma. Since updating is the dominant tendency in the task, this tendency must be suppressed to allow the maintenance of WM information by closing the gate.^13^ Altogether, longer RTs in former studies might therefor reflect in particular a more conservative decision criterion and a prolonged non-decision time. Especially the larger boundary separation parameter is of conceptual relevance, since it captures parts of the actual decision process during responding, while the non-decision time reflects processes outside of the decision process such as basic sensory processing and motor execution.^20^ The boundary separation reflects a threshold^20^ that must be reached until the decision process is completed (i.e., when to open or close the WM gate). The larger boundary separation during WM gate closing reveals that participants are more conservative during the decision,^20^ which in terms of an attractor model would mean that gate closing is reflected by a higher “bump” in the attractor landscape than gate opening. The threshold dependency of WM gating is conceptually relevant since this finding connects the process of WM gating to neurobiological concepts of WM in prefrontal cortex regions.^17^^,^^18^^,^^19^ Moreover, the finding of a WM gate closing threshold connects with conceptions stressing the need for a goal-directed balancing of behavior, according to which a specific threshold needs to be reached in order to get from one goal-state (e.g., maintenance) to another goal-state (e.g., updating).^14^^,^^15^^,^^16^ The importance of a threshold-dependent WM gating mechanism is also corroborated by the neurophysiological data: the boundary separation (a) was predictable by the effective non-linear and linear network connectivities in WM gate opening and closing. More particularly, the analysis (see Figure 5) showed that the boundary separation was equally well predicted in all frequency bands, while the prediction of the accumulation rate of evidence within a decision process (i.e., drift rate) was significantly worse, which is shown by generally higher non-linear estimation errors of (v) compared to (a) (see Figure 5). Of note, as seen in Figure 5, the drift rate was significantly better predicted in gate opening than closing within the beta frequency band. Given that the drift rate reflects the speed of evidence accumulation, it would be conceptually more important for gate opening compared to closing, because gathering evidence is only needed for WM updating and not WM maintenance. In sum, this would suggest that specifically the beta band activity modulates the differentiation whether to accumulate information or not. More importantly, the finding that the boundary separation (a) was predictable using each of the three analyzed frequencies shows that the threshold-dependent mechanism is supported by a multitude of cognitive operations reflected by the distinct frequency bands, which we discuss in detail in the following. As this was evident in both gate opening and closing, a threshold-dependent mechanism is the most important computational factor underlying WM gating dynamics, which is dependent on network communication. Regarding the cortical structures being part of this network, our analysis revealed brain regions (see Figure 4), which were previously associated with WM functioning and cognitive control which we discuss in the following. First, we discuss findings on gate closing, and then on gate opening.

### WM gate closing

Two different effective connectivity profiles were evident defined by distinct functional neuroanatomical networks.

First, linear and non-linear effective connectivity between the supplementary motor area (SMA; Brodman area [BA] 6) and the parahippocampal gyrus (PHG; BA 27, 28, 36, 37) was evident in the theta frequency band. Beyond motor control and sequence processing,^36^ the SMA is essential in WM maintenance and updating^37^^,^^38^^,^^39^^,^^40^ as well as cognitive flexibility (task switching)^41^ and has previously been shown to be involved in WM gating.^10^^,^^12^ The current finding that the SMA is only involved in gate closing is in line with the role of theta band activity in medial and superior frontal cortices to support cognitive control.^42^^,^^43^^,^^44^^,^^45^^,^^46^^,^^47^^,^^48^ Cognitive control is central for shielding information stored in WM, and this is what WM gate closing is about.^2^^,^^9^^,^^10^ Yet, to do so, there must be some connection to structures involved in storing WM content. Notably, the effective connectivity analysis revealed bi-directional connections to the PHG, known to be involved in the formation and maintenance of WM information.^49^^,^^50^^,^^51^ Taken together, it is possible that the PHG and SMA exchange information bi-directionally to enable gate closing, as this process relies on the contribution of memory-related areas that define and store a response rule (i.e., PHG) and areas involved in processes enabling a shielding of information through cognitive control and response selection according to rules stored in WM (i.e., SMA). Responses change consistently in the task and are not bound to the trial type itself but to the matching condition (i.e., left key for mismatch, right for match; see Figure S3 in the supplemental information). The activation of the SMA-PHG-network—specifically in the theta frequency band—may explain how relevant content information (reference vs. comparison) and their corresponding response pattern can be preserved in WM despite constantly changing responses/key-presses (match and mismatch) as theta plays an important role in WM control^52^ and modulates the sequential encoding of WM elements.^2^^,^^9^^,^^10^^,^^22^ Crucially, an even stronger effective connectivity pattern than in the theta band during gate closing was found in the beta frequency band.

In the beta band, the effective connectivity between the PHG and the lingual gyrus (LiG; BA 18, 19), which together with the PHG forms the medial occipitotemporal gyrus (MOG), is central. The LiG processes visual memories^53^^,^^54^^,^^55^ and is recruited especially for the maintenance of visual objects relevant to decide which task is to apply (e.g., X in red frame).^56^ Further, it is also associated with cognitive flexibility.^41^ Given that beta activity is associated with the monitoring, maintenance, and re-activation of WM content,^24^^,^^25^^,^^26^ the bi-directional effective connectivity of the LiG and the PHG likely enables the maintenance of visual information of object properties in WM. Of note, non-linear connectivity revealed a stronger directed communication from the PHG to the LiG than the other way around, which suggests that the PHG is the most central part in the beta activity network and controls processes in connected cortical structures during WM gate closing. It seems that beta activity is of specific importance when it comes to gate closing, as normalized connectivity was significantly greater in gate closing than opening only in beta, but not in theta frequency band (Figure 4). This suggests that gate closing depends to a greater degree on a strong network architecture than gate opening within the beta frequency band (see also discussion below on gate opening). It is the network connectivity that is important for WM information maintenance enabling a shielding against distractions by closing the gate. This is line with the notion that especially beta activity may be essential for maintenance of behaviorally relevant information during dynamic action control.^25^

### WM gate opening

As shown in Figure 4, during gate opening, normalized connectivity within the beta activity network was lower compared to gate closing. Conceptually, gate opening involves a reduction in the shielding of WM information to enable WM updating. As discussed earlier, the DDM showed that WM gating is threshold dependent, and this threshold is directly predictable by the strength of network connectivity. Therefore, the weaker network connectivity during WM gate opening could reflect a means to lower the threshold to update WM with new information. Concerning the brain regions involved, the supramarginal gyrus (SMG; BA 40), the superior frontal gyrus (SFG; BA 8), and regions of the pre (PrG; BA 1,2,3)- and postcentral gyrus (PoG; BA 4) (i.e., primary motor and somatosensory cortex, respectively) were present. These three regions revealed bi-directional connections implying an exchange of information between the areas. Thus, the network architecture and the functional neuroanatomical structures involved change, as compared to WM gate closing mechanism, to enable the processing of novel information. This shows that, depending on the necessities to either shield information and maintain it in WM (i.e., WM gate closing see earlier text) or flexibly update WM information (i.e., WM gate opening), brain structures specialized for either information maintenance or information updating and the flexible handling of information become involved. Indeed, all three bi-directionally connected brain regions involved in WM gating subserve flexible handling of information: the SMG is involved in flexible response selection^57^^,^^58^ and in WM processes like manipulation of WM information and monitoring of target events for WM updating.^28^^,^^57^^,^^58^^,^^59^ The SFG is critical for WM performance,^60^^,^^61^^,^^62^^,^^63^ whereby especially the lateral part of the SFG (BA 8) plays a role in the updating of WM content.^64^ Further, the SFG is associated with cognitive control and flexibility^65^ and SFG regions are especially involved in the change/switch of a response program based on updated rules.^66^^,^^67^ Therefore, the involvement of primary motor areas is also reasonable and also corroborates previous work on WM gating.^11^

Within the theta frequency band, and as with the beta frequency band, there was a change in the network architecture and brain regions involved, compared to WM gate closing processes useful to maintain information in WM. During WM gate opening, bi-directional connections between the inferior frontal gyrus (IFG; BA 44) and the PHG (BA 27, 28, 36, 37) were evident. As mentioned before, the PHG is involved in WM formation and this function is, of course, relevant during WM gate opening since new information passes the decision threshold (see DDM data) and updates WM content. Regarding the IFG, metanalytical evidence shows that especially the left IFG is implicated in WM^68^ and its activity scales with increases in WM load.^62^^,^^63^ Notably, the IFG plays a central role during interference control,^69^^,^^70^ which is important during WM updating in order to still be able to control for interfering information, which would otherwise compromise goal-directed actions. It is possible that the bi-directional connectivity between the IFG-PHG-network enables WM updating by controlling for possible detrimental effects of the new information to be integrated in WM content. This interpretation well fits to the general view in the literature according to which theta band activity is important for interference monitoring and its resolution.^42^^,^^43^^,^^44^^,^^46^^,^^47^^,^^48^ However, it is likely that the control of possibly interfering information during WM gate opening not only is a function of theta band activity but is also reflected by the alpha band activity.^25^

Opposed to WM gate closing, where only one region (see Table 1) was evident, two bi-directionally connected brain regions reflected alpha activity modulations during WM gate opening. These were the middle temporal gyrus (MTG; BA 21) and the inferior parietal lobule (IPL; BA 40). While the MTG is also involved in information maintenance in WM,^71^^,^^72^^,^^73^ it has recently, and using the same experimental approach, also been reported to be involved in WM gate opening processes.^12^ The reason for this is that the MTG is also involved in response selection and stimulus-response mapping processes^41^^,^^74^^,^^75^ and thus processes are informed during WM gate opening to enable goal-directed actions. Such an updating of information for response selection likely reflects a general function of inferior parietal regions,^12^^,^^76^ which also explains why the IPL was found important during WM updating.^1^^,^^77^^,^^78^ The bi-directional communication within the alpha band between the MTG and the IPL likely enables gate opening, with the MTG providing information about past response rules and thus the basis of what needs to be updated. This, likely, forms the basis for processes by the IPL regulating which information is gated into WM. This interpretation is well in line with the literature where alpha activity has been conceptualized to act as a “gatekeeper”^27^ controlling which information is used to inform goal-directed acting.^25^

### Limitations of the study

While the current study provides details and in-depth insights into the role of specific neural oscillatory activity in WM gating processes, it does not reveal insights into the causal relevance of the oscillatory activity and the associated functional neuroanatomical structures of WM gate opening and closing. This should be the focus of future studies using non-invasive brain stimulation approaches.

Further, some specific limitations of our approach need to be addressed. Even though the mathematical approach of source localization using LCMV has been tested many times, it has some obstacles. For example, we did not have individual brain scans from participants. Instead, we used a standardized brain model for the analysis, therefore ignoring the interindividual variance of brain structures within our sample. Additionally, electroencephalogram (EEG) source localization approaches can never achieve the accuracy of a magnetic resonance imaging (MRI) image. Nevertheless, LCMV beamforming offers a good opportunity to identify the approximate sources of neurophysiological activity measured with EEG, and cognitive science conceptual frameworks do not make predictions on a microscale level requiring high-resolution MRI data.

Furthermore, utilizing an artificial neural network in the nCREANN method to capture the relationships between the source signals requires a large amount of data for training and effectively fitting the multivariate autoregressive (MVAR) model to the signals. To achieve adequate data length for the analysis, single trials were concatenated in the present study. However, this makes training the network a time-consuming and computationally expensive procedure, especially for large datasets. Another factor that needs to be considered is that the current study estimates constant effective connectivity over time, considering the entire trial time interval. Estimation of time-varying connectivity may provide additional information about the dynamic system of WM gating, which will be addressed in our future work.

Finally, there are also some limitations regarding the investigation of WM gating itself, as it is only a theoretical concept and cannot be measured or manipulated directly. Hence, WM gating is conceptualized as a switch from maintaining to updating information (i.e., gate opening) and vice versa (i.e., gate closing). The reference-back task offers an indirect way to derive WM gating processes by subtracting maintenance and updating processes from the overall WM process. To date, it is the only WM task that allows investigating WM gate opening and closing on a behavioral level.

### Conclusions

Through computational modeling of behavioral data, we show that gate opening and closing are opposing threshold-dependent mechanisms. These WM gating (updating) thresholds are established by effective network connectivities in the theta, beta, and alpha frequency bands. It is shown that the directed network connectivity patterns predicted threshold-dependent WM gating mechanisms. Most important for threshold-dependent WM gating dynamics (i.e., WM gate closing and WM maintenance) is directed beta band parieto-frontal and parahippocampal-occipital network communications followed by theta network communication. For the latter, directed fronto-parahippocampal connectivities were observed. Both frequency bands play distinct roles during the maintenance of information in WM and shielding this information against possible interferences. For the latter, alpha band activity was also shown to be important, likely acting as a “gatekeeper” supporting the processes reflected by beta and theta band activity. The study shows that the decision criterion of whether to open or close the WM gate is established through the concerted interplay of directed communications in distinct neuroanatomical networks defined by beta and theta band activity.

## STAR★Methods

### Key resources table

**Table:** 

REAGENT or RESOURCE	SOURCE	IDENTIFIER
**Deposited data**		

Raw data behavior	This paper	https://osf.io/95m3e/
Raw data EEG	This paper	https://osf.io/95m3e/
Custom Code	This paper	https://osf.io/95m3e/

**Software and algorithms**		

MATLAB	https://de.mathworks.com/products/matlab.html	RRID:SCR_001622
BrainVision Recorder	https://www.brainproducts.com/productdetails.php?id=21	RRID:SCR_016331
EEGLAB	http://sccn.ucsd.edu/eeglab/index.html	RRID:SCR_007292
FieldTrip	https://www.fieldtriptoolbox.org	RRID:SCR_004849
Presentation	http://www.neurobs.com/	RRID:SCR_002521
SPSS	http://www-01.ibm.com/software/uk/analytics/spss/	RRID:SCR_002865

### Resource availability

#### Lead contact

Further information and requests for resources and reagents should be directed to and will be fulfilled by the lead contact, Christian Beste (christian.beste@uniklinikum-dresden.de)

#### Materials availability

This study did not generate new unique reagents.

#### Data and code availability

EEG data and behavioral data have been deposited at the Open Science Framework (OSF) and are publicly available at https://osf.io/95m3e/.

All original codes have been at the Open Science Framework (OSF) and are publicly available at https://osf.io/95m3e/.

Any additional information required to reanalyze the data reported in this paper is available from the lead contact upon request.

### Experimental model and study participant details

#### Participants

For this study, data from two previous experimental studies^7^^,^^12^ in healthy individuals of european ancestry were aggregated (*N* = 63 in total; 20 males, mean age 25.1 ± 4.6 years, all right-handers). All participants had normal or corrected to normal vision, normal hearing, and no history of neurological or mental illnesses. Every participant provided their written, informed consent. The Helsinki Declaration was followed throughout all procedures and studies were approved by the Ethic Commission of TU Dresden. Given that the analysis of the drift-diffusion parameters with the fast-dm toolbox (see below) is very sensitive to extreme fast or slow reaction times, an outlier correction of reaction times (±2 SD around the mean) was performed. Consequently, two participants were excluded blind to the results from the analysis because of extreme fast reaction times resulting in a final sample of *N* = 61 individuals (19 males, mean age 24.8 ± 4.5 years).

### Method details

#### Reference back task

The Reference Back Task follows the idea of the classical N-back Task and was developed to investigate WM processes like updating and maintaining information in the light of gating processes (i.e., gate opening and closing), which regulate the information flow to the WM.^8^^,^^11^^,^^12^ While gate opening is triggered when useful task-relevant information is detected (i.e., allows updating of WM information), gate closing is triggered by distraction information (i.e., facilitates maintenance of information within the WM). In a traditional N-back task, participants determine whether an identical stimulus was shown n trials prior. The Reference Back Task uses the same logic but extends it for the factor trial type (i.e., reference vs. comparison), which is indicated by a red or blue frame (for a detailed description, see^10^). In the Reference Back Task, participants see the letters X and O and must decide whether the shown letter is identical to the previous reference letter (indicated by a red frame) or not by pressing the left (i.e., mismatch) or right (i.e., match) button. If the current letter is shown in a blue frame (comparison), updating is not required as the reference does not change. Hence, if the current letter is shown in a red frame, participants must update the reference information in WM (for details, see Figure S3 in the supplemental information).

#### Drift diffusion model

A drift-diffusion model (DDM) was employed to estimate the characteristics of the selection process underlying the accuracy and reaction times.^20^^,^^21^ Briefly, a DDM considers a decision process between two alternatives to be a noisy accumulation of evidence until the evidence reaches a threshold for one of these alternatives.^20^^,^^21^ In the current study, these alternatives were the responses “match” vs. “mismatch”. As indicated in the introduction, the parameters of interest were the boundary separation (a) to estimate how conservatively the participants made their choice, the drift rate (v) to estimate the speed of evidence accumulation, and the non-decision time (t0) to separate decision and non-decision parts of the selection process. This estimation was done depending on the type of switching (switch vs. non-switch trials) and on the trial type (reference vs. comparison trials), as both factors may influence the response selection process.

The fast-dm 30 toolbox^79^^,^^80^^,^^81^ was used to estimate the parameters of the drift-diffusion model (https://www.psychologie.uni-heidelberg.de/projekt/fast-dm/). Only correct trials were included in the modeling procedure. Within each subject, trials with extremely fast or slow reaction times (±2 SD around the mean) were excluded. Subsequently, participants with extremely fast or slow mean reaction times (±2 SD around the group mean) or an extremely low or high number of trials per condition (±2 SD around the group mean) were excluded (for a description of the final sample, see above). Thresholds for the drift-diffusion model were defined as “match” (upper threshold) and “mismatch” (lower threshold). Parameters were estimated at the single-subject level by applying the maximum likelihood optimization criterion implemented in the toolbox. The precision was set to 4.0. The percentage of contaminants (p), the intertrial variability of the starting point (szr), and the intertrial variability of drift rate (sv) were set to zero, and the relative starting point zr was set to 0.5. The boundary separation (a), drift rate (v), and non-decision time (t0) were estimated as a function of the “switch” (non-switch vs. switch trials) and “trial type” (comparison vs. reference trials) conditions.

#### EEG recording and preprocessing

EEG data was recorded with 60 spatial equally distributed Ag/AgCI electrodes using a QuickAmp or BrainAmp DC/ExG amplifier (Brain Products GmbH, Gilching, Germany), respectively. The sampling rate was 500 Hz. The recorded EEG signals were preprocessed via Automagic pipeline^82^ and EEGLAB toolbox^83^ on MATLAB 2020b (The MathWorks) with the following steps. First, the raw EEG signals were downsampled to 256 Hz. Next, EOG channels were identified (i.e., Fp1, Fp2, AF7, AF8) and flat EOG channels were removed. All the remaining channels were re-referenced to their average value and the removed EOG channels were interpolated using neighboring channels. After that, the preprocessing pipeline “PREP”^84^ were applied to remove the line noise of 50 ± 2 Hz or 100 ± 2 Hz. It was also used to detect noisy channels or channels with extreme amplitudes meeting any of the following criteria: more than 5 standard deviations away from the channel population; with low correlations (<0.4) with any other channel within 1 s; lack of predictability by other channels within 5 s (<0.75), containing noise higher than 5 standard deviation from the signal-noise ratio. After this data cleaning, a robust average reference was calculated. Following the “PREP” pipeline, an EEGLAB clean_rawdata function was utilized to detrend the EEG signal. This function separated out low-frequency signals using an IIR high pass filter of 0.5 Hz (stop band attenuation: 80 dB; transition band: 0.25 to 0.75 Hz). Besides that, it detected noisy channels that exceeded 4 standard deviations of the total channel population. Subsequently, EEG epochs with abnormally strong power were reconstructed using Artifact Subspace Reconstruction.^85^ After that, the flat channels were removed, and unrepaired time windows were identified as bad signals. Following that a low-pass FIR filter of 40 Hz with an order of 86 was applied.^86^ Artifacts from heart, muscle, and eye movements were classified and removed by an independent component analysis. Lastly, the discarded channels were interpolated via a spherical method. The cleaned EEG signals were segmented into epochs locking to the stimulus onset. Each epoch starts from 2000 ms prior to the stimulus and ends at the 2000 ms after stimulus presentation. Each epoch was normalized based on an average amplitude of baseline window (−200–0 ms relative to the stimulus). The obtained epochs were further categorized into four conditions as in described in the reference-back task based on the stimulus markers: switch-reference, nonswitch-reference, switch-comparison, and nonswitch-comparison.

#### Time frequency decomposition

The segmented EEG trials underwent time-frequency decomposition. As we aimed to focus on the theta (4–7 Hz), alpha (8–12 Hz), and beta (13–30 Hz) frequency bands, we employed a Morlet wavelet transformation approach to extract the time series of frequency bands ranging from 1 to 30 Hz with a step of 0.5 Hz using the FieldTrip toolbox.^87^ The length of the Morlet wavelet was 3 and the width was 5.5. The decomposed time-frequency representations were decibel-normalized by baseline activities from −200 to 0 ms relative to the stimulus onset. These decibel-normalized time-frequency representations were averaged across trials for each condition for each participant, and further averaged across respective frequency bands for theta, alpha, and beta activities. To examine effects of the experimental manipulations, we implemented cluster-based permutation tests for each frequency band. Specifically, we compared the switch-reference and nonswitch-reference conditions to examine the WM gate opening signature and compared the switch-comparison and nonswitch-comparison to examine the closing signature, separately. All cluster-based permutation tests were conducted as follows: First, a within-subject t-test were applied on the two conditions for each time point and electrode; neighboring data points with an alpha value of ≤0.05 were clustered. Then, a permutation test with 1,000 random draws using the Monte Carlo method were applied to establish a reference distribution and identify the clusters. The final t-values of the clusters were calculated by summing all t-values within the corresponding clusters.

#### Linearly constrained minimum variance beamforming

After extracting the time series for theta, alpha, and beta bands, we applied LCMV for each frequency band to construct the source of frequency activities from the sensor level data. This was done for each frequency band (theta, alpha, and beta) and each WM gating process (opening and closing) separately. We used the data from switch-reference and nonswitch-reference conditions to examine WM gate opening dynamic and the data from switch-comparison and nonswitch-comparison conditions to examine WM gate closing dynamic. First, a leadfield was calculated using a FieldTrip template “standard_bem” as head model. Unlabeled and cerebellum positions in the leadfield were removed. Second, the data from relevant conditions and respective frequencies were appended and further averaged and used to generate the covariance matrix. The covariance matrix was used to create a common filter for reconstructing the time series at the source level. Later, the common filter was applied to the time series of each relevant condition to construct the corresponding source activities. We contrasted the source activities of nonswitch trials from the switch trials following the Equation 1 as the gating effects (gate opening and closing, separately) at the source level (see above). Then, we identified the voxels showing the highest difference between contrasted conditions (1%) and clustered the selected voxels using a DBCAN method.^88^ The minimum number of voxels to form a cluster was 2. The source information including the voxel index and the cluster index were further used to extract the source signal for each cluster in each gating process. Due to the necessity of using single trial data as input in the nCREANN, we used the averaged source signal across trials in the nonswitch condition as the reference/baseline power for each voxel. Subsequently, each switch trial was subtracted by the baseline signal for each voxel. Therefore, the single-trial source signal of WM gating in each cluster were averaged from included voxels.

#### Effective connectivity analysis

To prepare the data for nCREANN, a Hamming windowed sinc FIR filter was applied on preprocessed EEG data to filter signals for the alpha, theta, and beta frequency bands, separately. This returned a time series with the same structure of the preprocessed EEG data for each frequency band. The resulting time series in each frequency band were then segmented and categorized into four conditions separately, and the time courses of their underlying sources were extracted in the next step. To analyze the effective connectivity in the present study, we utilized the machine learning-based approach nCREANN^32^ to assess the interactions among the brain regions in the theta, alpha, and beta frequency bands between the clusters created by the DBSCAN algorithm. The nCREANN is based on a non-linear Multivariate Autoregressive (nMVAR) model and employs an artificial neural network (ANN) to evaluate effective connectivity across brain regions. In contrast to simplified linear techniques, which exclusively consider linear MVAR models, the nCREANN methodology captures both linear and non-linear dynamics which has been shown to be crucial for the organization of information flow across cortical regions.^89^^,^^90^ An nMVAR process models the (non-linear) interactions between the past activity of the regions to construct their current samples. This concept tries to depict temporal causality, in which an effect cusses the future. For a given time series $x\left(n\right)\in R^{M}$ of length L, a non-linear MVAR model of order p is defined as

(Equation 2) $$x\left(n\right)=f\left(x_{p}\right)+\sigma \left(n\right)$$

Where $x_{p}={\left[x_{1}\left(n-1\right),x_{2}\left(n-1\right),\cdots ,x_{M}\left(n-p\right)\;\right]}^{T}$ is the vector of p past samples of (M) multivariate time series. The noise vector, $\sigma \left(n\right)={\left[\sigma_{1},\sigma_{2},\ldots ,\sigma_{M}\;\right]}^{T}\text{,}$ is a real valued zero-mean white noise vector, and the non-linear function f(.) quantitatively describes how the p previous samples cause the future values. In the nCREANN method, the functions f is divided into linear and non-linear part

(Equation 3) $$f=f^{Lin}+f^{NonLin}$$

and based on the $f^{Lin}$, the Linear Connectivity $\left({lC}_{i\to j}\right)$ is computed as the linear impact of ith cluster on the jth cluster, and based on the information embedded within $f^{NonLin}$, the Non-linear Connectivity, $\left({NC}_{i\to j}\right)$, is inferenced to establish the extent of the non-linear causal effect of $x_{i}$ on $x_{j}$.

In the present study, the nCREANN was applied to the time courses of the LCMV-derived sources in the gate opening and closing conditions. The data points of the trials in the time interval from −500 to 1500 ms were considered for the connectivity analysis. For training the network, all of the single-trial source signal values of WM gating were concatenated in order to have a sufficient data length. The optimum model order (p=10) was estimated with Akaike and Schwartz criteria^91^ and considered the same for all subjects in both conditions. For each subject, the connectivity values were normalized to be ranged in [0,1], by dividing them to the maximum value across all frequency bands of both conditions for each linear and non-linear connectivity measure. The connectivity patterns are shown with the connecting arrows (showing the information flow from one cluster to another one) whose thicknesses and their arrowhead sizes are proportional to the connectivity values. The non-self-connections were plotted for the average values across all subjects.

### Quantification and statistical analysis

#### Integration of network connectivity and DDM data

To examine the relationship between the neurophysiological and behavioral data, we used an artificial neural network approach to perform a non-linear regression between the connectivity information and the DDM parameters in the two gating conditions. The analysis was performed using “Function Approximation and Nonlinear Regression” toolbox in MATLAB2022. This method has been previously applied to assess the non-linear dependence of parameters, regardless of the nature of the relationship between them. In the present study, for each frequency band in gate opening and gate closing conditions, a one-hidden layer feedforward neural network was trained with the linear and non-linear connectivity values (averaged across all non-self-connections) for all subjects as the input, and the DDM parameters as the output. For comparing the Goodness of Fit of the non-linear regressions for each behavioral data the predictability of the new (unseen) DDM parameters based on the connectivity values was evaluated by a 10-fold cross-validation technique. To avoid the scaling of the behavioral parameters, the mean square of the estimation error of each of them was divided by the average values of their original amount.

#### Other statistical analyses

IBM SPSS Statistics 29.0.0.0 was used for the behavioral data analysis. We calculated error percentages (EPs) and averaged reaction times (RTs) for every participant, factor combination (i.e., switch-comparison-match, nonswitch-comparison-match, etc.) and gating process (i.e., gate opening and gate closing). Further, repeated-measures ANOVAs with the factors *Switching* (switch vs. nonswitch), *Trial Type* (reference vs. comparison), and *Matching* (match vs. mismatch) were used to analyze behavioral results (i.e., RTs and EPs) as well as DDM parameters (i.e., boundary separation, drift rate, and non-decision time). Post-hoc paired sample t-tests were used.
