# Supplementary material for: Neurophysiological effective network connectivity supports a threshold-dependent management of dynamic working memory gating
Source: iScience. 2024 Mar 18;27(4):109521. doi: 10.1016/j.isci.2024.109521 (PMC11000016; doi:10.1016/j.isci.2024.109521)
Supplement: Document S1. Figures S1–S3 [file mmc1.pdf]

iScience, Volume *27*

Supplemental information

Neurophysiological effective network connectivity supports a threshold-dependent management of dynamic working memory gating

Julia Elmers, Shijing Yu, Nasibeh Talebi, Astrid Prochnow, and Christian Beste

Supplementary Material

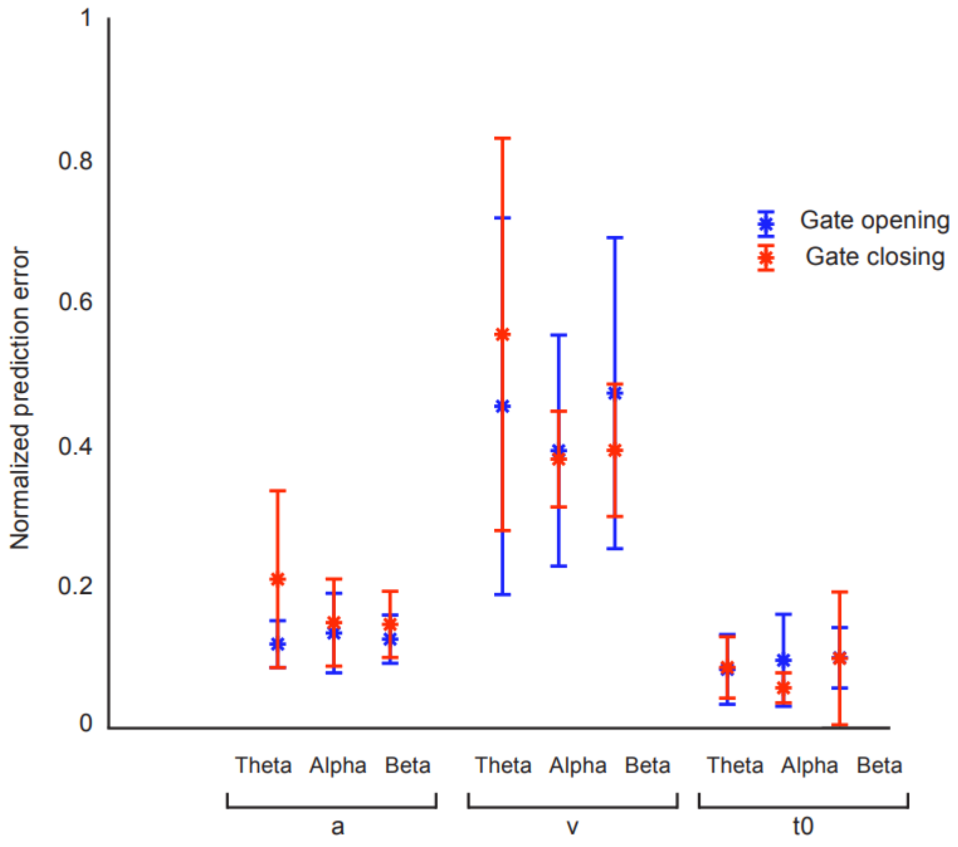

Figure S1. Non-Linear Regression Analysis. Results of non-linear regression analysis with normalized prediction errors based on neuronal activity index (NAI) value. Based on the NAI value, all estimation errors overlap between the gate opening and gate closing conditions, and there is no significant difference in the estimation of the parameters.

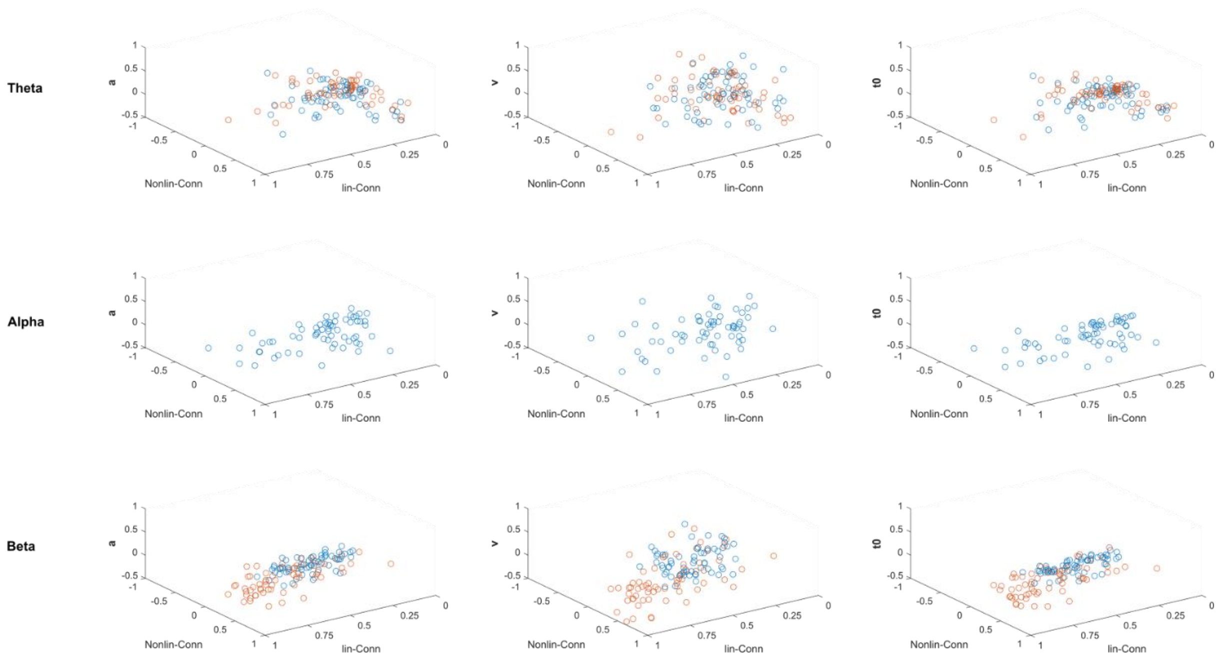

Figure S2. Relationship between effective connectivity and DDM measures. Scatter plots of the linear connectivity (Lin-Conn), nonlinear connectivity (Nonlin-Conn), and the DDM parameters (a, v, t0) for theta, alpha, and beta frequency bands show a complex and non-linear distribution of the connectivity/DDM parameters.

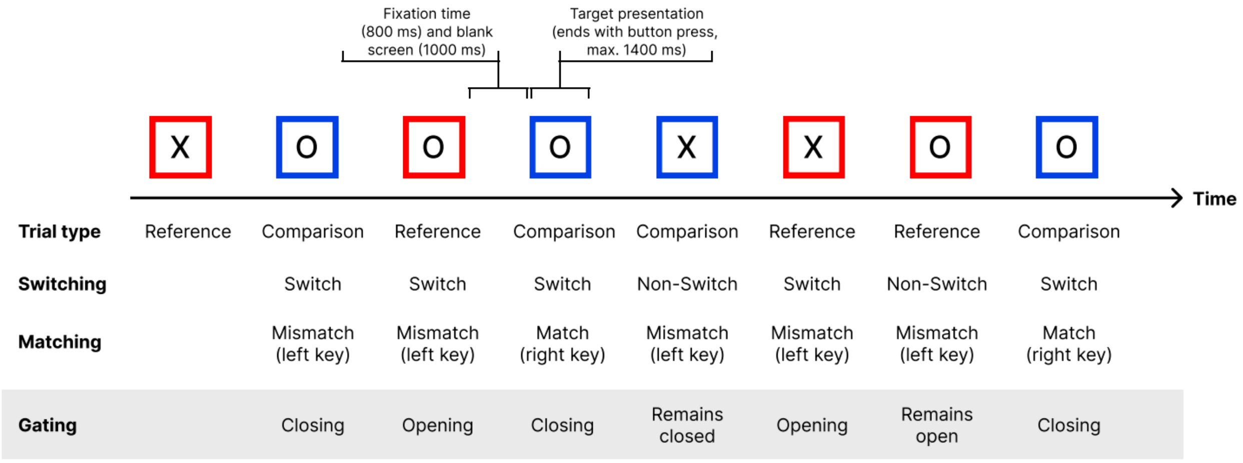

Figure S3. Reference Back Task. Example of a reference back task (excerpt). In each trial, participants must decide whether the shown letter is identical to the previous reference letter (indicated by a red frame) or not by pressing the left (i.e., mismatch) or right (i.e., match) button. A trial consists of a fixation time (800ms), followed by a blank screen (1000 ms), and the target presentation (up to 1400 ms). Gate closing is described by the switch from a reference to a comparison trial, while gate opening occurs when switching from a comparison to a reference trial. In trial repetitions, the gate remains closed (i.e., repetition of comparison trials) or open (i.e., repetition of reference trials).
